# Origin and Meaning of the Term “Biology”

**Published:** 1894-01

**Authors:** 


### Origin and Meaning of the Term "Biology,"

The word "biology," which is now so familiar as comprising the sum of the knowledge which has yet been acquired concerning living nature, was unknown until after the beginning of the present century. The term was first employed by Treviranus, who proposed to himself as a like task the development of a new science, the aim of which should be to study the forms and phenomena of life, its origin and the conditions and laws of its existence, and embodied what was known on these subjects in a book of seven volumes, which he entitled *Biology, or the Philosophy of Living Nature.* He desired to found the new science on the fundamental distinction between living and non-living material. In discussing this distinction, he takes as his point of departure the constancy with which the activities which manifest themselves in the universe are balanced, emphasizing the impossibility of excluding from that balance the vital activities of plants and animals. The difference between vital and physical processes he accordingly finds, not in the nature of the processes themselves, but in their co-ordination—that is, in their adaptness to a given purpose, and to the peculiar and special relation in which the organism stands to the external world. He defined life as consisting in the reaction of the organism to external influences, and contrasts the uniformity of vital reactions with the variety of their exciting causes. This idea suggests "the idea of organism" as that to which all other biological ideas must relate. It also suggests, although perhaps it does not express it, that action is not an attribute of the organism but of its essence—that if, on the one hand, protoplasm is the basis of life, life is the basis of protoplasm; their relations to each other are reciprocal. We think of the visible structure only in connection with the invisible process.

The origin of life—the first transition from non-living to living—is a riddle which lies outside our scope. No seriously-minded person, however, doubts that organized nature as it now presents itself to us has become what it is by a process of gradual perfecting advancement, brought about by the elimination of those organisms which failed to obey the fundamental principle of adaptation which Treviranus indicated. Each step, therefore, in this evolution is a reaction to external influences, the motive of which is essential as that by which, from moment to moment, the organism governs itself. And the whole process is a necessary outcome of the fact that those organisms are most prosperous which look best after their own welfare. As in that part of biology which deals with the internal relations of the organism, the interest of the individual is in like manner the sole motive by which every energy is guided We may take what Treviranus called selfish adaptation—*Zweckmassigkeit fur sich selber*—as a connecting link between the two branches of biological study. Out of this relation springs another, which, I need not say, was not recognized until after the Darwinian epoch—that, I mean, which subsists between the two evolutions, that of the race and that of the individual.

Biology naturally falls into three divisions, and these are even more sharply distinguished by their methods than by their subjects, namely: Physiology, of which the methods are entirely experimental; morphology, the science which deals with the forms and structure of plants and animals, and of which it may be said that the body is anatomy, the soul development; and, finally, œcology, which uses all the knowlege it can obtain from the other two, but chiefly rests on the exploration of the endless varied phenomena of animal and plant life as they manifest themselves under natural conditions.—J. Burdon Sanderson, M.D., in *British Med. Journal.*

